# Let’s make it personal: CRISPR tools in manipulating cell death pathways for cancer treatment

**DOI:** 10.1007/s10565-024-09907-z

**Published:** 2024-07-29

**Authors:** Mobina Bayat, Javid Sadri Nahand

**Affiliations:** Infectious and Tropical Diseases Research Center, Tabriz University of Medical Sciences, Tabriz, 15731 Iran

**Keywords:** Cancer, Cell death, CRISPR screening, CRISPR-Cas, Personalized therapy

## Abstract

Advancements in the CRISPR technology, a game-changer in experimental research, have revolutionized various fields of life sciences and more profoundly, cancer research. Cell death pathways are among the most deregulated in cancer cells and are considered as critical aspects in cancer development. Through decades, our knowledge of the mechanisms orchestrating programmed cellular death has increased substantially, attributed to the revolution of cutting-edge technologies. The heroic appearance of CRISPR systems have expanded the available screening platform and genome engineering toolbox to detect mutations and create precise genome edits. In that context, the precise ability of this system for identification and targeting of mutations in cell death signaling pathways that result in cancer development and therapy resistance is an auspicious choice to transform and accelerate the individualized cancer therapy. The concept of personalized cancer therapy stands on the identification of molecular characterization of the individual tumor and its microenvironment in order to provide a precise treatment with the highest possible outcome and minimum toxicity. This study explored the potential of CRISPR technology in precision cancer treatment by identifying and targeting specific cell death pathways. It showed the promise of CRISPR in finding key components and mutations involved in programmed cell death, making it a potential tool for targeted cancer therapy. However, this study also highlighted the challenges and limitations that need to be addressed in future research to fully realize the potential of CRISPR in cancer treatment.

**Graphical abstract:**

Current application of CRISPR system in cancer therapy through a glance. **A** choosing the appropriate biological model for screening in vitro (using established cell lines, animal derived tumor cells, human derived tumor cells, stem cells or T cells), in vivo (using animal models which can harbor human derived tumor), or ex vivo (human/animal-derived organoids). **B** preparation of CRISPR gRNA library. **C** experimental design of CRISPR screening, identification of the desired gRNAs or phenotypic response. **D** CRISPR-Cas targeting of the identified targets, with Cas9 gene editing system (Knockout, base editing, prime editing), RNA modulation (modulation of RNA splicing, RNA base editing, RNA interference), and epigenomic edits and CRISPR interference/activation using dead Cas9 (dCas9) (Bock et al. 2022b)

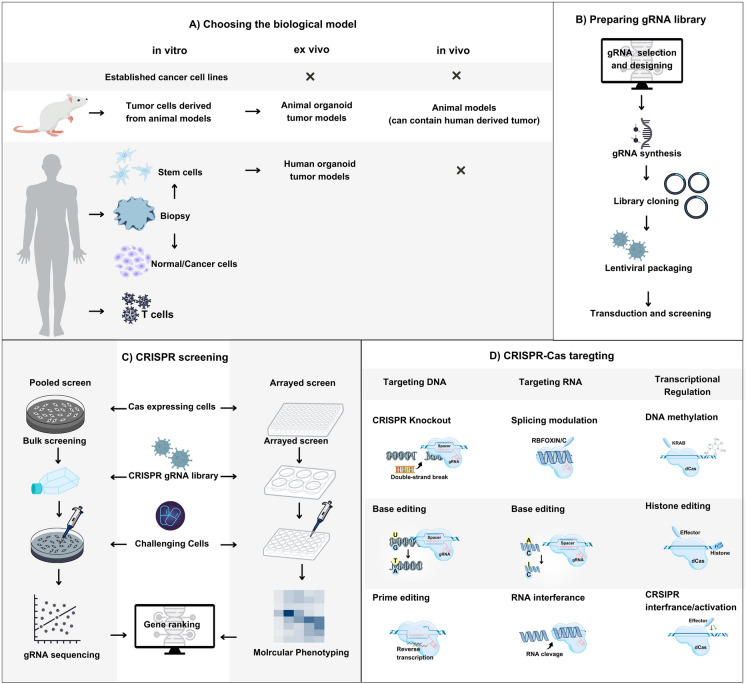

## Introduction

CRISPR (Clustered Regularly Interspaced Short Palindromic Repeats), once considered as the bacterial immune system for defeating invasive viruses, is now an ineluctable tool in biological studies (Adli 2018). Jennifer Doudna and Emmanuelle Charpentier, pioneers of revolutionary CRISPR gene editing were the Nobel laureates in chemistry 2020 (Ledford and Callaway 2020). On December 8th, 2023 the U.S. Food and Drug Administration permitted the first CRISPR treatment for sickle cell disease in patients 12 years and older (Adminstraion USFaD. 2023). This powerful technology has appeared as a marvelous genome editing tool in cancer research due to its programmability and flexibility (Chow and Chen 2018). CRISPR screens are a great source of biological discovery that enables to identify the relationship between genotype and phenotype and gene functions in diverse tumor biologies such as metastasis, cell death, and therapy resistance (Bock et al. 2022a; He et al. 2021). In fact, in recent years CRISPR screens have been greatly adopted for evaluating cell death pathways and modulators in human cells and various diseases such as Parkinson’s disease, Alzheimer’s disease, and cancer (Pavlou et al. 2023; Malireddi et al. 2023; Kuehnle et al. 2023). A genome-wide CRISPR-Cas9 death screen has also been introduced for positive selection and deep sequencing of dying cells (Arroyo et al. 2016). CRISPR-Cas9 can also be employed for treating c by repairing mutations or knocking out (KO) precise genes. Abundant researches have been carried out on tumor therapy in related fields. Personalized and targeted therapy using CRISPR-Cas9 system has vastly shaped the development of tumor treatment (Jiang et al. 2020a). Therefore, ever since its adaptation for mammalian cells, CRISPR has engendered a vigorous tool for altering most aspect of cell function (Katti et al. 2022).

Cell death is one of the critical cellular processes which is broadened to all aspects of life, including homeostasis, development, and immune regulation of multicellular organisms (Duprez et al. 2009; Kist and Vucic 2021). Several forms of programmed cell death have been classified over the last decades including apoptosis, necroptosis, pyroptosis, and autophagic cell death, all of which relying on a diverse subset of proteins for the activation and execution of their respective pathway (Duprez et al. 2009; Kist and Vucic 2021). During the development of cancer, normal cellular growth processes are disrupted, and the natural safeguards that normally eliminate abnormal cells through apoptosis are disabled. As a result, tumor cells are unable to undergo programmed cell death (apoptosis) and instead, they die through necrosis, a process characterized by the loss of cellular integrity and eventual cell death. This phenomenon provides a promising alternative therapeutic approach, as targeting and inducing necrosis in cancer cells may be a more effective way to eliminate tumors than traditional apoptosis-based therapies. Necrosis in apoptosis-defected cancer cells occurs once the rate of energy consumption exceeds the rate of energy production. Therefore, the autophagic catabolic process is demanded by cells to provide an alternative energy source during metabolic deprivation to avoid necrosis. Considering the critical role of autophagy in survival of these cells, autophagy also appears as a suitable therapeutic target in cancer (Jin et al. 2007). On the other hand, pyroptosis, a programmed necrotic cell death (Kong 2023), creates a chronic inflammatory environment for tumorigenesis that exerts a protumor effect and accelerates the immune escape of tumors. Pyroptosis participates in both tumorigenesis and antitumor immunity at all stages of tumor development, depending on the timing, the tumor type, host inflammatory and immunity state, and implicated effector components all of which needs to be closely controlled (Jia et al. 2023). Ferroptosis is a recently elucidated form of regulated cell death, distinguished by the presence of iron overload, the accumulation of lipid-derived reactive oxygen species (ROS), and lipid peroxidation. This unique mode of cell death exhibits distinct morphological, biochemical, functional, and transcriptional profiles that differentiate it from other forms of RCD, including necrosis, apoptosis, and autophagy. Emerging research suggests that ferroptosis plays a significant role in the initiation, progression, and regulation of cancer, highlighting its potential as a therapeutic target for cancer treatment (Wang et al. 2020).

Altogether, highlights the importance of screening the cell death pathways and targeting the individual mutations for efficient and personalized cancer treatment. Therefore, considering the wide use of CRISPR technologies, in cancer research particularly through regulation of programmed cell death, we aimed to review the very recent approaches for screening and modifying cell death associated genes in order to elaborate the important role of CRISPR in personalized cancer research.

## CRISPR tools from screening to targeting

Over the past three decades, the prokaryote-derived CRISPR–Cas genome editing systems paid a magnificent contribution to manipulating, detecting and editing specific DNA and RNA sequences in mammalian cells. Several studies have been dedicated for understanding the biology and development of CRISPR technologies for diverse research areas including fundamental science and translational medicine (Katti et al. 2022; Pickar-Oliver and Gersbach 2019). Personalized cancer therapy has been defined as treatment based on the molecular features of a tumor from an individual subject, and exhibits a remarkable capacity for the treatment of several cancer types (Wistuba et al. 2011). Development of CRISPR–Cas technology as accessible and programmable tools for screening these molecular characteristics and identifying the best therapeutic targets is accelerating a revolution in cancer treatment. Paired with the speedy development of reference and individualized genome sequence information, CRISPR–Cas based approaches are paving the way of limitless genetic manipulation (Fellmann et al. 2017).

**Fig. 1:**
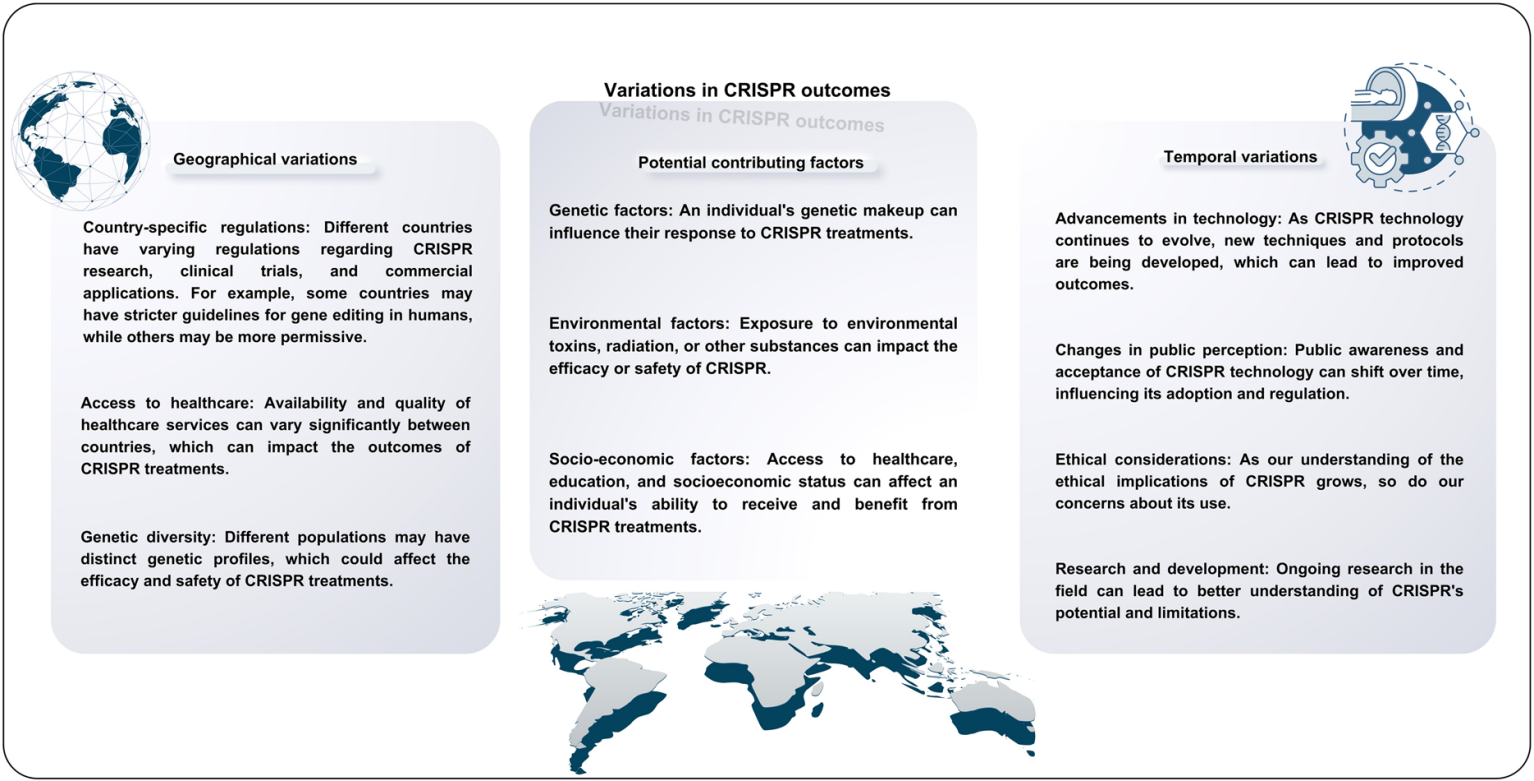
Variations and challenges that affect CRISPR outcomes. The successful application of CRISPR technology is not uniform across all regions and has shown varying outcomes depending on the country, continent, and time frame. Several factors contribute to these differences, including genetic, environmental, and socio-economic factors

As illustrated in Fig. 1***,*** there are several variables within the using the CRISPR systems that can affect the outcome. In different cell types, targeting different genes, have different consequences. This sentence simply explains the foundation of CRISPR tools. In CRISPR screens different sets of gRNAs (guide RNA) are designed to target different genes in cells in order to observe their phenotypic consequences. After finding the desired phenotypic results, the CRISPR-Cas gene editing tools can be used to target the identified genes to achieve the given outcome. Obviously, this process requires different steps, optimizations, and faces with several challenges and limitations. Therefore, through this section, we shortly introduce the application of CRISPR tools from screening to targeting in order to highlight their protentional in precise and personalized cancer treatment.

### CRISPR screens

CRISPR screens provide a powerful and flexible foundation for biological discovery, as well as unbiased exploration of gene function in various applications (Bock et al. 2022a). CRISPR screening has unraveled numerous molecular mechanisms in basic biology, immunology, medical genetics, and cancer research (Bock et al. 2022a). Currently several screen methods exist, such as pooled CRISPR screen, arrayed CRISPR screen, high-content CRISPR screens, all have been detailly discussed previously (Bock et al. 2022a, 2022b). Using pooled CRISPR screens, which involves analyzing the presence or absence of specific genetic modifications, researchers have discovered genes involved in various biological processes, such as regulating gene expression, controlling epigenetic markers, signaling pathways, and cell growth. Most CRISPR screens target one single gene per cell; this simplifies data analysis but make the evaluation of gRNA efficiency, off-target effects and etc., more difficult (Bock et al. 2022a). CRISPR screens are widely utilized in cancer research given the high availability of models and the cancer relevance of readily screenable phenotypes including cell proliferation and drug resistance (Bock et al. 2022a*).*

In order to start with CRISPR screening, the first step is choosing the appropriate biological model (e.g., cell lines, organoids, and animal models) to capture the desired biological process (***Graphical abstract 1A***). In that context, in vivo CRISPR screens provide high-throughput interrogation of multifaceted processes in cancer (Chow and Chen 2018). However, most of CRISPR screens in cancer research have been performed in vitro using established cell lines or cellular transplant models, which are not in native tissue microenvironment involving complex interactions of multiple cell types. Whereas in vivo studies are of importance for evaluating the physiologic relevance of identified targets and to faithfully recapitulate the development of human cancer (He et al. 2021; Lin et al. 2022). Furthermore, organotypic models of patient-specific tumors have transformed the view on cancer heterogeneity and the application of personalized medicine. This is attributed to the capability of organoid models to stably preserve genetic, proteomic, morphological and pharmacotypic features of the parent tumor in vitro, while also providing unprecedented genomic and environmental manipulation (LeSavage et al. 2022). After choosing the adequate model, it is critical to choose the appropriate Cas endonuclease (Cas9, AsCAs12a, LbCAs12a, Cas13a, dCas, and etc.) and gRNA (***Graphical abstract B***).

Obviously, the timing is of critical importance and can vastly affect the results of CRISPR screens. In screens for critical genes, for instance, read-outs at early time points mostly identifies genes whose KO results in immediate cell death (genes implicated in transcription and translation), while later time points could detect genes that are indirectly influencing cell proliferation and fitness (Bock et al. 2022a). After screening gRNAs and their targets are ranked based on their effect on the desired phenotype with existing software packages (MAGeCK, CRISPY, DrugZ, and CERES). Afterwards, the identified top hits are evaluate in terms of plausibility and biological relevance (***Graphical abstract C***) (Bock et al. 2022a).

On the other hand, pooled CRISPR screening based on fluorescence-activated cell sorting (FACS) is a vigorous forward genetic tool for investigating complicated signaling networks. CRISPR-Cas9 can be exploited as a powerful approach for the detection of cellular cascades that control the fate of a specific target protein when merged to a phenotypic selection step by FACS. Therefore, it represents a great potential in targeting disease-causing candidates such as oncogenes (DeJesus et al. 2016).

As a powerful tool, CRISPR screening has enabled us for high-throughput identification of unique gene functions through linking specific genetic modifications to subsequent phenotypes. Also, pooled CRISPR screens made it feasible to discover innate and adaptive immune response regulators in the setting of cancer and infection (Holcomb et al. 2022). CRISPR screening is a great tool for identification of targets for regulating different types of cell death. For instance, CRISPR screening has been used for identifying regulators and mediators of necroptosis in fibroblast cells (Callow et al. 2018). This technology has revealed multiple suppressors of ER stress response implicated in avoiding apoptosis and restoring homeostasis (Panganiban et al. 2019; Chidawanyika et al. 2018). Therefore, CRISPR screening has provided a powerful tool for monitoring cell death, identifying the vulnerabilities, detecting the mutations, recognizing the implicated pathways, and the most suitable targets in cancer models both in vivo and in vitro*.*

### CRISPR attacks

Genome editing technology has been described as modification of intracellular DNA (e.g., integrations, insertions, deletions, and sequence substitutions) in a sequence-specific manner (Moon et al. 2019). As one of the magnificent scientific discoveries of this century, the CRISPR-Cas system have provided an RNA-guided genome engineering platform for precise genome modifications in living eukaryotic cells (Yang et al. 2021). The variety of CRISPR-Cas enzymes has expanded the ability of CRISPR-based platforms (Hsu et al. 2014; Zhan et al. 2019). Cas9 is a DNA endonuclease capable of inducing double strand breaks (DSBs) at specific genomic loci. The CRISPR-Cas13a system can be programmed for knockdown of mRNAs and long non-coding RNAs at specific sites. Meanwhile, more studies are required to demonstrate the potential of this system for treating or diagnosing human genetic diseases (Li et al. 2020). Another example is Cas12a that simplifies the process of target multiplexing through the ability to catalyse the maturation of its own gRNAs (Katti et al. 2022). The catalytically inactivated Cas9 protein known as dead Cas9 (dCas9) is able to bind to the target DNA but unable to create DSBs. Fusion of the dCas9 to modifying enzymes or transcriptional regulators enable targeting transcriptional and epigenome machinery (Holcomb et al. 2022). Moreover, dCas9 coupled with a custom gRNA, which binds to the complementary DNA elements, is capable of exerting transcriptional control through halting the elongation by RNA polymerase and subsequently repressing the target gene (Larson et al. 2013). The inactivated Cas9-based transcriptional control that inhibits the initiation and elongation of transcription through complementary base pairing with the target sequences is termed as CRISPR interference (CRISPRi). Also, it is plausible to exploit CRISPRi for controlling multiple genes at the same time via customizing the sgRNA (Cui et al. 2017; Zhang et al. 2016). Furthermore, CRISPRi based on dCas9 is capable of repressing transcription in mammalian cells, while fusion of the dCas9 to transcriptional repressor domains elevates its effect (Kampmann 2018). For instance, CRISPRi is able to inhibit the transcription of target genes through combining the Krüppel-associated box (KRAB) repressor with the specific DNA recognition of dCas9 (Gilbert et al. 2013) (***Graphical abstract 1D***). It has been demonstrated that targeting gene repression with the CRISPRi system results in reduced cell apoptosis in CHO cells. Also, it has been shown that different repressor fusion types can improve the CRISPRi system to elevate the gene repression efficiency (Xiong et al. 2019).

Despite the canonical use of targeting this system for KO of an exact genome loci, CRISPR has been reengineered for various aims such as base editing, DNA methylation, transcriptional regulation (activation and repression), histone modification, and genome architecture manipulation. CRISPR-Cas9 screens have revealed a comprehensive landscape of genes implicated in cancer biology, encompassing regulators of PD-L1 expression, determinants of drug resistance, and key players in synergistic and synthetic lethal interactions, as well as other mechanisms driving tumorigenesis (Chow and Chen 2018).

**Fig. 2:**
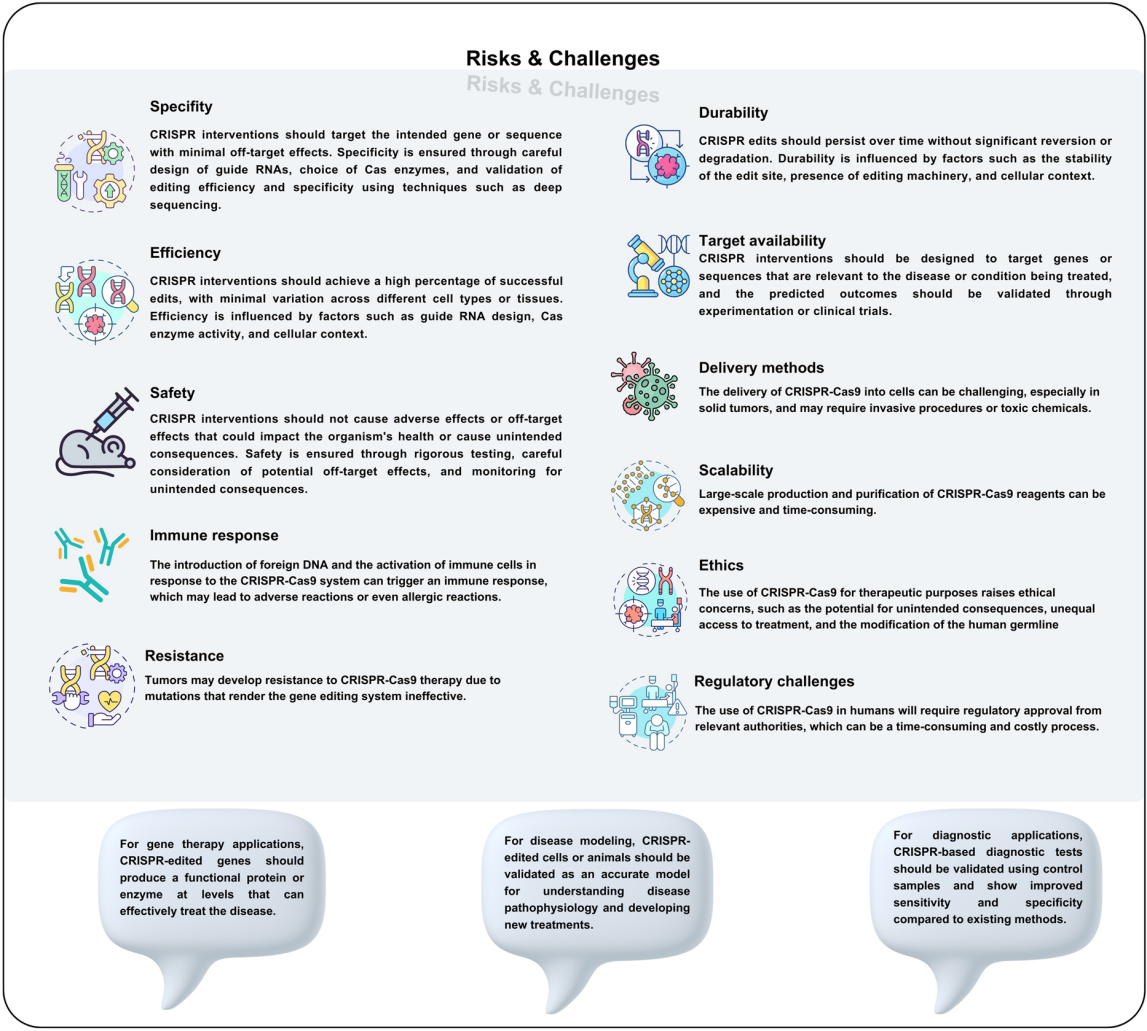
Criteria for Evaluating CRISPR Interventions. Evaluating CRISPR interventions requires a multifaceted approach, considering various criteria to assess their efficacy, safety, and potential impact

Considering its efficiency in precise targeting, CRSIPR-Cas gene editing tools has been widely used in targeting cell death pathways in cancer cells, through targeting regulatory proteins and non-coding RNAs (Aquino-Jarquin 2017; Eslami et al. 2023). Figure 2 illustrates the criteria for evaluating the success and accuracy of CRISPR interventions. In addition, these tools have been successfully used for identifying different mutations and regulating the expression of molecular components that are implicated in different cell death pathways. In that regard, considering the promise of CRISPR applications in targeting tumor cell death, we dedicated the next section we review the application of CRISPR tools in different type of regulated cell deaths in cancer.

## Cell death pathways in *Cancer*: CRISPR’s good deeds

### Apoptosis: the enchant

**Fig. 3:**
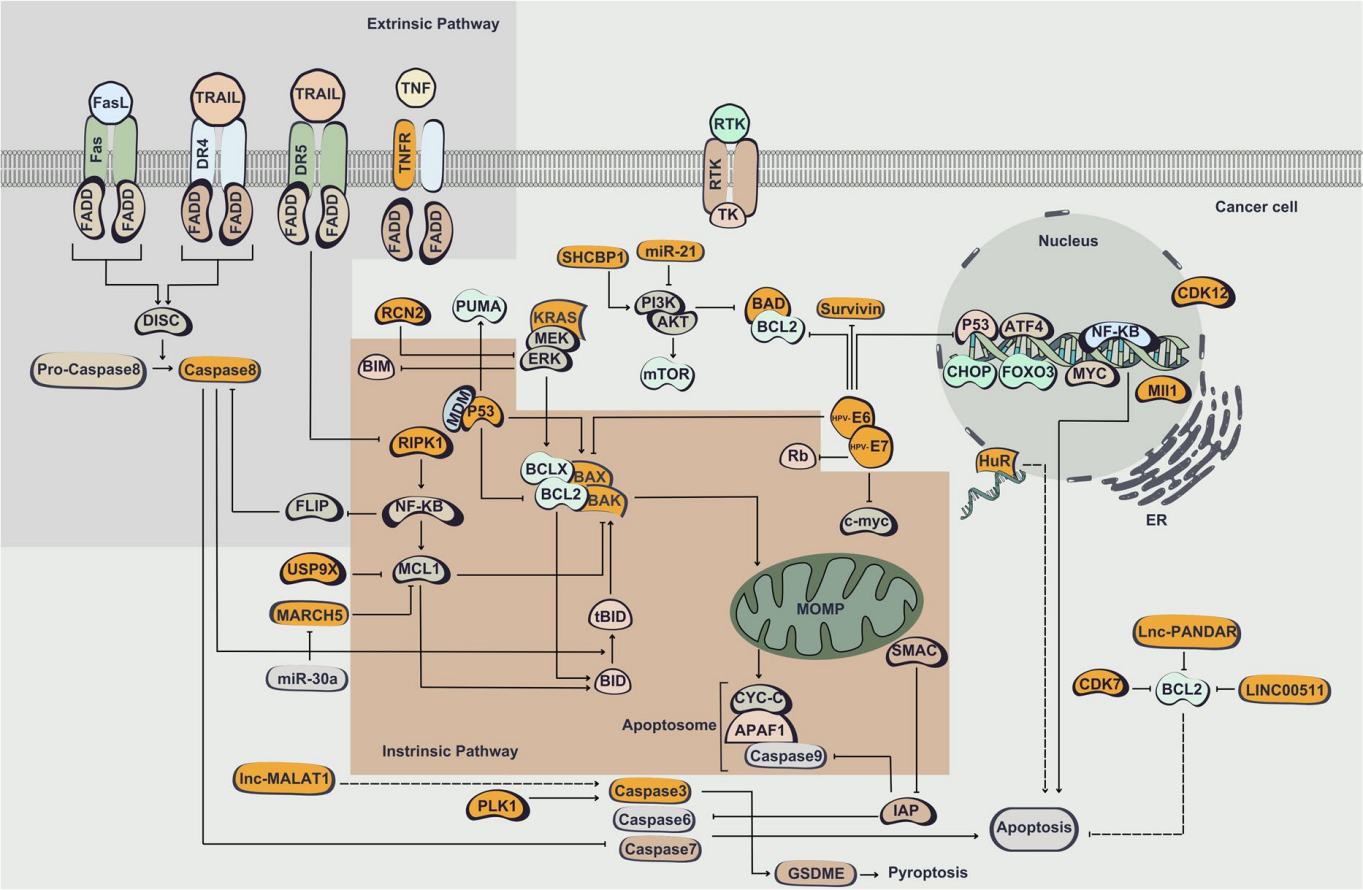
CRISPR-Cas targeting of intrinsic and extrinsic apoptosis pathway in cancer. The extrinsic pathway initiates once death ligands attached to a death receptor. Death receptors recruit adapter proteins such as TNF receptor-associated death domain (TRADD), Fas-associated death domain (FADD), and caspase 8. This Binding of the death ligand to the death receptor results in death-inducing signaling complex (DISC) formation. DISC then activates caspase 8, which initiates apoptosis through cleavage of downstream or executioner caspases. the intrinsic pathway is initiated by intracellular stimuli and is regulated by Bcl-2 family. BIM and BID stimulate the oligomerization of effectors BAX and BAK to induce mitochondrial outer membrane permeabilization (MOMP), release of cytochrome c (CYC-C), which activates caspase 3 through forming apoptosome and eventually cell death occurs. The components that have been targeted by CRISPR system in cancer cells are represented in yellow

Apoptosis is a programmed intrinsic suicide mechanism accelerated by executioner caspases that leads to the regulated cell breakdown into apoptotic bodies, which are then recognized and surrounded by phagocytes (Duprez et al. 2009). Two distinct pathways of apoptosis have been identified: the intrinsic and extrinsic pathways. As represented in Fig. 3 the extrinsic pathway is triggered by extracellular stimuli, such as Fas and tumor necrosis factor (TNF), which activate initiator caspase-8/10 through the formation of a death-inducing signal complex (DISC). In contrast, the intrinsic pathway is initiated by the activation of BH3-only proteins, including BID, which is cleaved by caspase-2 or -8/10 into tBID. Through their interaction with antiapoptotic BCL-2 family proteins or direct binding, BH3 proteins induce the activation of multi-BH domain proteins, ultimately leading to mitochondrial outer membrane permeabilization (MOMP). This process releases proapoptotic factors such as SMAC and cytochrome c into the cytoplasm (Fernald and Kurokawa 2013).

Dysregulation within extrinsic and intrinsic pathways result in evasion of apoptosis, and consequently therapy resistant (Noori et al. 2023; Raudenská et al. 2021; Singh and Lim 2022). The disruption of crucial apoptosis regulators, including P53, BAX, and BAK can lead to the evasion of programmed cell death and the development of cancer. This occurs when cells become transformed, exhibit genomic instability, and acquire stem-like properties through c-Myc-dependent transcriptional regulation. (Godwin et al. 2021). As a result, instead of undergoing apoptosis in response to therapy or other selective pressures, resistant cells can proliferate, disseminate, and eventually decrease patient survival (Carneiro and El-Deiry 2020). In this context, CRISPR-Cas tools holds a great promise in apoptosis induction (Rahimi et al. 2019). In fact, this system has served greatly in screening the mutations and components implicated in apoptosis resistance of cancer cells as well as targeting the identified candidates for promoting apoptosis in cancer cells. For instance, using CRISPR/Cas9 screen in A549 cells has revealed that DSBs in the protein Hu antigen R (HuR) encoding gene induced apoptosis (Gao et al. 2022). This shows the CRISPR screens are also able to identify anti-apoptotic genes as targetable mediators of chemo-resistance (Stover et al. 2019a). Therefore, this system has been used in identification and targeting of critical genes in order to sensitize cancer cells to apoptosis induced by chemotherapy agents such as imatinib (Jung et al. 2021), navitoclax (Jung et al. 2021), NCB-0846 (Jung et al. 2021), cisplatin (Stover et al. 2019b), and paclitaxel (Stover et al. 2019b).

Recent CRISPR-Cas9 screens have identified cell type-specific vulnerabilities in cancer, including acute myeloid leukemia (AML), both in vivo and in vitro. In orthotopic xenograft models, CRISPR screening has shown that MARCH5 plays a critical role in preventing apoptosis and that its repression enhances the effectiveness of BCL-2 inhibitors including venetoclax (Lin et al. 2022). MARCH5 was also found to be upregulated in breast cancer cells and to contribute to the poor survival rate (Tang et al. 2019). Therefore, suggesting *MARCH5* as a clinically potential therapeutic target in cancer therapy.

CRISPR screening has also identified cyclin-dependent kinase 12 (CDK12) as an essential target for regulating apoptosis and cell survival in prostate cancer (Lei et al. 2021). Moreover, CRISPR-based KO screening has identified that depletion of *MIEF2* promoted oxaliplatin resistance in colorectal cancer through suppressing the mitochondrial apoptosis cascade in colorectal cancer organoids (Xie et al. 2022). In fact, organoids are being widely utilized for studying regulated cell death pathways in various cancers (Csukovich et al. 2024; Schmitt et al. 2022; Shen et al. 2022; Shi et al. 2022).

Despite screening apoptosis pathways in cancer cells, the application of CRISPR-Cas gene editing has been a great contributor in promoting tumor cell apoptosis. For instance, Cas9 has been successfully programmed for KO of *HuR* gene and initiation of anti-proliferative signaling pathways, apoptosis, necrosis, and subsequent cell death via enhancing the level of associated mRNA and proteins in tongue squamous carcinoma cell (Wang et al. 2021). Survivin is another protein functional in the modulating cell cycle progression and apoptosis inhibition, encoded by Human Baculoviral inhibitor of apoptosis repeat-containing 5 (*BIRC5*). Using CRISPR-Cas9 for *BIRC*5 gene KO in leukemic cells elevated cell death and apoptosis level (Jubair and McMillan 2017). Furthermore, CRISPR-Cas technique can be utilized for generating apoptosis-resistant cell lines through dampening pro-apoptotic genes, including *caspase-3,6,7*, and *Alf1* (Zhang et al. 2017). The core effectors of apoptosis include caspase proteolytic enzymes (Boatright and Salvesen 2003). In fact, caspases serve a crucial role in the induction, amplification, and transduction of intracellular apoptotic signals (Fan et al. 2005). Mounting evidence have suggested that caspase-3 elevates stress induced cancer cell growth, migration, invasion, and tumor angiogenesis (Zhou et al. 2018). The binding of cytochrome C to apoptotic protease activating factor-1 (APAF-1) triggers the formation of an apoptosome complex. This complex then interacts with pro-caspase-9, leading to its cleavage and subsequent activation of caspase-3 (Sarkar et al. 2023). As alluded in Fig. 3***,*** CRISPR-Cas technology has been used for targeting caspase-3 in colorectal cancer cells (Zhou et al. 2018). In fact, elevated activity of caspase-3 has been established as a sign of apoptosis and a positive indicator of efficacy in cancer treatment (Zhou et al. 2018). Furthermore, the caspase-3 can switch between apoptosis and pyroptosis in cancer through interacting with gasdermin E (GSDME) (Jiang et al. 2020b).

The identification of cell type-specific vulnerabilities and the regulation of apoptosis in cancer provides new avenues for the development of targeted therapies using the CRISPR-Cas system.

CRISPR-Cas system has also been used for targeting non-coding RNAs and viral oncoproteins that are implicated in apoptosis regulation. For instance, CRISPR-Cas9 gene KO of long non-coding RNA *UCA1* in ovarian cancer cells exhibited some positive effects in eliminating cancer cell proliferation and elevating the apoptosis through upregulating *FAS*, *BAK*, *BAX*, and *P53* genes and downregulating *BCL-2* and *SURVIVIN* genes (Montazeri-Najafabadi et al. 2022). In addition to Cas9, Cas13a has also been used in cancer research for programmed RNA manipulation (Zhao et al. 2018; Palaz et al. 2021). LncRNA gastric cancer associated transcript 3 (GACAT3), is greatly involved in apoptosis regulation and development of various cancers including cancers of breast, colorectal, bladder, glioma and more importantly gastric (Su et al. 2021; Yuan et al. 2022). Targeting GACAT3 in human bladder cancer T24 and 5637 cell lines using CRISPR-Cas13 resulted in inhibited cell proliferation and increased apoptosis (Zhang et al. 2021).

On the other hand, human papillomavirus (HPV), a well-known oncovirus, is associated with all cervical cancer’s cases, as well as several other cancers such as prostate cancer (Sadri Nahand et al. 2020; Khatami et al. 2022), lung cancer (Hussen et al. 2021; Rezaei et al. 2020), head and neck cancers, especially oropharyngeal cancer (Szymonowicz and Chen 2020). HPV contains critical oncoproteins including E6 that interacts with P53, surviving, and Bax/BCL-2 pathways, and E7 that interact with retinoblastoma (Rb) and c-myc (Khatami et al. 2022). Therefore, both oncoproteins are characterized as apoptosis modulators (Khatami et al. 2022; Amjad et al. 2023; Najafi et al. 2022). The loss of either E6 or E7 viral proteins expression results in cell-cycle arrest and apoptosis. Also, the retinoblastoma protein (pRb) pathway promotes cell senescence, and the re-establishment of this protein is associated with deactivation of E7 gene expression. Besides, reactivation of the P53 pathway due to E6 protein repression results in senescence and apoptosis (Jubair and McMillan 2017).

Using the CRISPR/Cas9 system to specifically target and disrupt the E6 and E7 oncogenes in cervical cancer cell lines infected with HPV16 and HPV18. The result was the induction of apoptosis and inhibition of cell growth, indicating a potential therapeutic approach for treating HPV-positive cervical cancer. Disruption of the E6 and E7 genes which is tailed with the loss of the respective oncoproteins can restore the P53 expression and subsequently the tumor suppressor pRb.

The findings suggest that utilizing a gRNA specific to the HPV16-E7 gene may be a promising therapeutic strategy for treating cervical cancer. This approach has the potential to effectively target and disrupt the E7 oncogene, which is a key driver of tumorigenesis in HPV16-positive cervical cancer. (Jubair and McMillan 2017; Hu et al. 2014).

CRISPR systems have also provided an opportunity for targeting cancer-causing mutations that result in apoptosis deregulation. For instance, direct targeting of mutant K-Ras variants with CRISPR approaches led to a significant suppression in cell viability and proliferation in vitro, along with tumor growth suppression in vivo (Bender et al. 2021). CRSIPR-Cas targeting of K-Ras has also occurred in organoid models. For instance, CRISPR-Cas9-mediated targeting of K-Ras in colorectal cancer organoids has resulted in induced apoptosis and reduced cell viability (Boos et al. 2022). In addition, the effect of mutant K-Ras knockdown, via CRISPR exerts regulatory effects on the downstream molecules such as ERK, PI3K, AKT, c-Myc, and STAT3 (Bender et al. 2021). The study suggests that after K-Ras-driven cellular transformation, therapeutic strategies can target crucial signaling events that enable tumorigenesis. This can be achieved by blocking key pathways, such as K-Ras, ERK-1/2, JNK, VEGF/VEGFR2, AP-1, and Nrf2/anti-apoptotic factor translation. Additionally, allowing the regulation of mitochondrial apoptosis by P53 and BAX-p18 can also be an effective approach to induce apoptosis and prevent tumor growth (Godwin et al. 2021). In that regard, components of K-Ras cascade have been targeted by CRISPR based strategies more often than K-Ras itself. However, despite all the promise, more investigations are required to establish treatment strategies based on targeting K-Ras mutations with CRISPR-Cas9 system (Bender et al. 2021). More studies are alluded in Table 1.

**Table 1:** Using CRISPR system for targeting programmed cell death pathways (PCD) in cancers

Cancer	Target	Model	PCD type	Note	Ref
Lukemia	*BIRC5*	In vitro (KG1 and HL60 cell lines)	Apoptosis	Promoted apoptotic cell death	Yao et al. 2024)
Cervical Cancer	HPV16-*E7*	In vitro (HeLa)	Apoptosis	CRISPR-Cas9 targeting of E6 and E7 results in the induction of p53 or Rb, cell cycle arrest and cell death in cervical cancer cells	Kennedy et al. 2014)
Breast cancer	SHCBP1	In vitro (MCF-7 and MDA-MB-231 cell lines)	Apoptosis	CRISPR-Cas9 knockout of SHCBP1 increased cyclin-dependent kinase inhibitor p21, and decreased the Cyclin B1 and CDK1	Feng et al. 2016)
Glioblastoma	*PLK1*	In vitro (in 005 (murine GBM) and OV8 (human ovarian carcinoma) cells)	Apoptosis	CRISPR-Cas9 targeting of *PLK1* caused tumor cell apoptosis, suppressed tumor growth, and enhanced survival in orthotopic GBM	Rosenblum et al. 2020)
Colorectal cancer	*KL*	In vitro (Caco-2)	Apoptosis	Overexpression of the klotho (*KL*) gene using CRISPR-Cas9 system results in elevated apoptosis and suppressed cell motility in cancer cells	Soykan and Gunes 2024)
Small-cell lung cancer	*CDC7*	In vitro (H69-AR cells)	Apoptosis	Silencing serine/threonine kinase cell division cycle 7 (*CDC7*) using CRISPR/Cas9 system in chemo-resistant SCLC cells decreased the IC_50_ and improved the efficacy of chemotherapy	Deng et al. 2023)
Prostate Cancer	*Lcn2*	In vitro (PC3 cells)	Apoptosis	Lcn2 knockout with CRISPR-Cas system decreased cell proliferation, migration, and increased CDDP-induced apoptosis in PC3 cells	Rahimi et al. 2019)
Breast cancer	*OPN*	In vitro (MDA-MB-231)	Apoptosis	CRISPR-Cas9 targeting of osteopontin (OPN) along with radiation results in elevated apoptosis, downregulation of downstream genes, suppressed cell viability, and inhibited cell-cycle progression in breast cancer cells	Ghanbarnasab Behbahani et al. 2023)
Prostate cancer	Androgen receptor (AR)	In vitro (LNCaP cells)	Apoptosis	Using CRISPR/Cas system for disrupting the AR inhibits the growth of androgen-sensitive prostate cancer cells	Wei et al. 2018)
Bladder cancer	*ERIC*	In vitro (T24 and 5,637 cells)	Apoptosis	Upregulation of ERIC using CRISPR-dCas9-VPR results in inhibition of cell proliferation, Invasion and promotion of apoptosis in bladder cancer	Yang et al. 2021)
Cutaneous melanoma	*CDK2*	In vitro (A375 cells)	Apoptosis	Knockout of Cyclin-dependent kinase (CDK) using CRISPR/Cas9 system results in induction of G0/G1 phase arrest and apoptosis in A375 melanocytes	Liu et al. 2020)
Melanoma	*Atg5*	In vitro (A375P)	Autophagy	CRISPR-Cas9 targeting of ATG5 resulted in absence of LC3 modification and increased resistant to gossypol in melanoma cells	Kim et al. 2016)
Lung adenocarcinoma	*Atg7/ Atg13*	In vitro (U1810)	Autophagy	Knockout of *Atg7* and *Atg3*, results in reduction of ULK1 protein expression and eventually leads to autophagy blockage	Allavena et al. 2016)
Non-small cell lung cancer	*NNT*	In vitro (A549 (A549/DDP) cells)	Autophagy	Altering DNA methylation of nicotinamide nucleotide transhydrogenase (*NNT*) using CRISPR system rescued the cisplatin resistance of lung cancer cells through reduction of autophagy	Xu et al. 2023)
Colorectal cancer	*miR-34a* and *miR-34b/c*	In vitro (HCT116)	Autophagy	The simultaneous deletion of *miR-34a* and *miR-34b/c* Using CRISPR-Cas9 system suppressed proliferation after p53 activation, enhanced migratory, invasive, and EMT capacities, as well as increased resistance to chemotherapy-induced apoptosis and an augmented autophagic response upon treatment with 5-fluorouracil (5-FU). Additionally, the deletion of these microRNAs led to a reduction in apoptosis and an upregulation of autophagy-related gene expression	Huang et al. 2023)
Breast cancer	*Nsfl1c (*p47 encoding gene*)*	In vivo (Female athymic nude mice), In vitro (N148 cells)	Autophagy	CRISPR knockout screen identified p47 as a metastasis inhibitor in HER2 + breast cancer through regulation of NEMO trafficking and autophagy flux In vivo	Hao et al. 2024)
Nasopharyngeal carcinoma	*LUC7L2*	In vitro (CNE2IR cells)	Autophagy	Using a CRISPR/Cas9 high-throughput screening have identified *LUC7L2* as a promoter of radioresistance via autophagy in nasopharyngeal carcinoma cells. Also, knockdown of *LUC7L2* in combination with autophagy inhibitor, chloroquine (CQ), leads to a more NPC-radioresistant cell death	Shen et al. 2021)
Anaplastic thyroid cancer	*MADD*	In vitro (8505C cells)	Autophagy	Deletion of MADD using CRISPR-Cas9 system can induce autophagy through downregulating MAPK and PI3K/AKT/mTOR signaling in anaplastic thyroid cancer	Bakthavachalam et al. 2024)
Cervical cancer	*STAT3*	In vivo (four weeks old female nude mice), In vitro (SiHa and Hela cells)	Autophagy	Using CRISPR-Cas9 system for overexpression of STAT3 results in downregulation of LC3B. STAT3 knockout or knockdown significantly elevates autophagy and decreases proliferation, migration, plate colony formation and subcutaneous tumorigenesis both in vivo and in vitro	Wu et al. 2022)
Pancreatic cancer, Breast cancer, Cervical cancer	*USP48*	In vivo (C57/B6 mice), In vitro (HeLa, MCF7, and Panc-1 cells)	Pyroptosis	Using CRISPR-Cas9 screen have identified that loss of USP48 inhibits cell pyroptosis	Ren et al. 2023)
Gastric cancer	*GSDME*	In vitro (SGC-7901)	Pyroptosis	Knockout of *GSDME* by CRISPR-Cas9 switched 5-FU induced pyroptosis into apoptosis in gastric cancer	Wang et al. 2018)
Lung Cancer	*STK11*, *KEAP1*	In vivo (Mice), In vitro (H358 and H292 cells)	Ferroptosis	STK11/KEAP1 Co-mutation enhances proliferation In vitro and tumor growth In vivo	Wohlhieter et al. 2020)
Endometrial carcinoma	*ADCK3*	In vitro (Ishikawa cells)	Ferroptosis	CRISPR-Cas9 targeting of ADCK3 suppresses MPA-mediated ferroptosis by abrogating arachidonate 15-lipoxygenase (ALOX15) transcriptional activation	Zhang et al. 2023)
AKT-hyperactivated cancer	*TRPML1*	In vitro, In vivo	Ferroptosis	CRISPR-Cas9 targeting of TRPML1 gene or disruption of its interaction with ARL8B protein has been demonstrated to inhibit cancer cell growth both in vitro and in vivo, and enhance sensitivity to ferroptosis	Zhang et al. 2024)
Ovarian cancer	HIF-1α	In vitro (ES-2 cells)	Ferroptosis	HIF-1α-depletion by CRISPR diminishes the sensitivity to ferroptosis in ES-2 cells	Zou et al. 2019)

Furthermore, using CRISPR interference (CRISPRi) in CHO cells to repress the endogenous expression of apoptotic genes BAK, BAX, and caspase-3 led to reduced apoptosis and caspase activity, along with enhanced mitochondrial membrane integrity (Xiong et al. 2019). Overall, CRISPR tools hold a great promise in cancer therapy through identifying the vulnerabilities, the source of apoptosis resistances, inducing apoptosis and/or sensitizing cells to apoptosis induced by chemo- or radio-therapy.

### Autophagy: a classic

Autophagy is a conserved catabolic cascade that enables eukaryotic cells to degrade cytoplasmic components in response to pleiotropic extra- and intra-cellular stimuli (Levine and Kroemer 2008; Liang and Corn 2022). The dysregulation of autophagy is correlated with the pathogenesis of several serious conditions and a therapeutic intervention in diseases, such as cancer (Onorati et al. 2018).

**Fig. 4:**
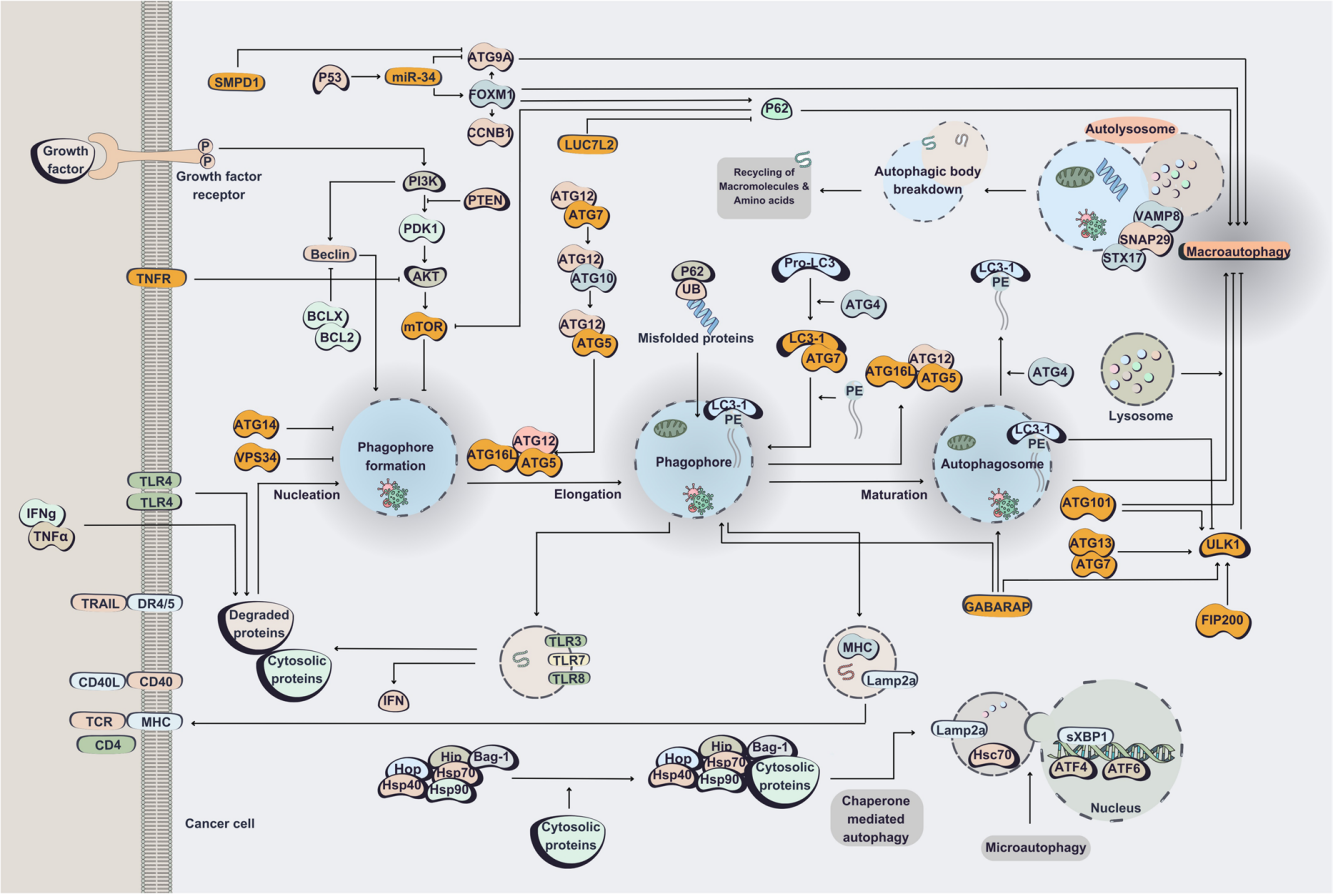
CRISPR-Cas targeting of autophagy pathway in cancer. The components that have been targeted by CRISPR system in cancer cells are represented in yellow. During macro-autophagy, Beclin-1/VPS34 control the phagophore formation in response to stress signaling pathways, Atg5–Atg12 conjugate and interact with Atg16L to form phagophore. After processing and insertion of LC3 into the phagophore membrane and formation of autophagosome it fuses with lysosome and forms autolysosome to recycle the up taken components. In micro-autophagy, cytosolic components are directly up taken by the lysosome itself. In chaperone-mediated autophagy, targeted proteins are unfolded and degraded through their translocation across the lysosomal membrane in a complex with chaperone proteins (e.g., Hsc-70) and their subsequent recognition by the lysosomal membrane receptor lysosomal-associated membrane protein 2A (LAMP-2A)

Autophagy plays a crucial role in the degradation and recycling of cellular components, and its dysregulation has been implicated in the development and progression of cancer, as well as the interaction between tumors and their surrounding stroma. Furthermore, autophagy is a key regulator of the tumor microenvironment and cellular response to chemotherapy, influencing the effectiveness of cancer treatment (Li et al. 2019). Furthermore, suppression of apoptotic proteins in cancer, can shift a cellular stress response from apoptosis to autophagy (Raudenská et al. 2021). Therefore, autophagy appears as a promising target for cancer treatment and its induction in response to therapy can be considered as having a pro-death or a pro-survival role (Sui et al. 2013). Thus, understanding the novel function of autophagy could result in development of a therapeutic strategy to elevate the clinical outcomes in cancer treatment. A genome-wide CRISPR screen was employed to reveal the macroautophagy pathway, thereby elucidating the constituent components of the initiation, nucleation, and elongation phases of the autophagic process. (DeJesus et al. 2016). Recent data also demonstrated that chemotherapy resistance is associated with the activation of autophagy (Pagotto et al. 2017). In fact, autophagy is established to elevate drug resistance by suppressing apoptosis and promoting metastasis (Qin et al. 2023). Autophagy modulators such as core autophagy-related (*ATG*) genes can also counteract cancer drug resistance (Qin et al. 2023). As described previously (Mizushima 2020) and alluded in Fig. 4,* ATG* encoded proteins are the main modulators in autophagy process. In that regard, CRISPR tools have been outstanding candidates in identifying and precise targeting of the molecular machinery involved in autophagy related therapy resistance in cancer cells. For instance, using a CRISPR-Cas9 to generate *ATG5* KO ovarian cancer stem cells have led to clarification of the pivotal role of autophagy in cancer stem cells maintenance. Furthermore, suggesting autophagy inhibition in combination with chemotherapeutic applications as a sufficient strategy to overcome drug resistance and tumor recurrence (Pagotto et al. 2017).

While autophagy is considered as a pivotal process in some tumor cells, it is unclear if or how these cells can circumvent the suppression of autophagic cascade. With that context, CRISPR-Cas9 has been used for targeting *ATG7* in autophagy-dependent cells, lung cancer (H292 cell lines), breast cancer (BT549 cell lines), and colon cancer (HCT116 cells) cells. This study established that these cells are not even able to survive with complete genetic inactivation of *ATG7* and multiple other autophagic regulators, but they are also able to resist pharmacological autophagy inhibition probably by overexpressing NRF2, which is required to circumvent *ATG7* KO and maintain protein homeostasis (Towers et al. 2019). Furthermore, KO of the *ATG7* in human erythroleukemia cells (K562 cell line) with CRISPR-Cas9 resulted in defected canonical autophagy which is associated with attenuated cell cycling, proliferation and differentiation potential through the failure of ATG5-ATG12 conjugation and LC3-I/LC3-II conversion while maintaining their ability for apoptosis (Wang et al. 2016).

In fact, ATG5 has been the center of attention for cancer treatment due to its implication in controlling cell proliferation, migration, invasion and the tumor immune microenvironment, which affects radio- and chemo-therapy resistance, along with the overall patient survival (Zhou et al. 2023a). In that regard, CRISPR-Cas9 KO of *ATG5* in A375P cell line resulted in lack of autophagy and reduced LC3-II. The Cas9 targeting *ATG5* triggers non-homologous end joining and leads to the total ablation of ATG5 protein expression (Kim et al. 2016). CRISPR-Cas9 has also been used for KO of *ATG5* and generation of the intestinal IPEC-J2 autophagy-deficient cell line. This study suggested that utilzing the double nicking CRISPR-Cas9 system rather than CRISPR-Cas technology pose more advantages for minimizing off-target activity (Tang et al. 2015). Furthermore, CRISPR-Cas application has been successful in targeting autophagy regulatory signaling cascades, including but not limited to AMP-activated protein kinase (AMPK) signaling cascades (Towers et al. 2019; Grenier et al. 2018), the phosphatidylinositol 3-kinase/mammalian target of rapamycin (PI3K/mTOR) (Dai et al. 2021; Sui et al. 2013), and tumor necrosis factor α (TNF-α) (Cai et al. 2019, Farhang et al. 2017). For instance, using CRISPR-Cas system for targeting the tumor necrosis factor receptor (TNFR) in colorectal cancer cells, established an approach for impairing proliferation and colony formation in vitro which is associated with the suppression of protein kinase B or Akt (PKB/Akt) and induction of autophagy-induced cell death (Li et al. 2023a).

Non-coding RNA-mediated autophagy can alleviate drug resistance (Qin et al. 2023). As alluded in Fig. 4 the *miR-34* (*miR-34a/b/c*) encoding genes are directly targeted by the P53 transcription factor, and are mediators of the P53 tumor inhibitory effects. CRISPR-Cas9 targeting of *miR-34a* and/or *miR-34b/c.*

in the HCT116 colorectal cancer cell line induces proliferation following p53-mediated activation, which enhances stress-induced autophagy, suppresses apoptosis, and upregulates autophagy-related genes after 5-FU treatment. In contrast, individual knockout of *miR-34a* or *miR-34b/c* has less significant effects on these processes. Simultaneous knockout of both *miR-34a* and *miR-34b/c* impairs p53-DREAM-mediated gene repression and further enhances autophagy after 5-FU treatment. In cells with *miR-34a/b/c* deficiency, overexpressed FOXM1 directly induces p62 and ATG9A, leading to increased autophagy, apoptosis suppression, and resistance to 5-FU. Autophagy suppression via *ATG9A* knockout or chloroquine re-sensitizes miR-34-deficient cells to 5-FU treatment (Huang et al. 2023).

Furthermore, upregulation of gasdermine B (GSDMB), a member of the Gasdermin (GSDM) genes, in HER2 breast cancer promotes poor prognosis, aggressive, and resistant to anti-HER2 agents through elevating protective autophagy. It has been established that under specific conditions, GSDMs can trigger pyroptosis pro-inflammatory cell death (Gámez-Chiachio et al. 2022). *GSDMB* KO using CRISPR-Cas9 targeting approach in HCC1954 and OE19 cells resulted in suppressed autophagic flux and subsequently enhanced cytotoxic effect of lapatinib therapy in contrast to wild cells.

The co-localization of LC3B and Rab7 was found to be reduced in GSDMB-knockout cells following lapatinib plus chloroquine treatment, suggesting that GSDMB is involved in the formation of an autophagic protein complex. Notably, GSDMB upregulation confers resistance to anti-HER2 agents in HER2 cancer cells by promoting protective autophagy. Mechanistically, the N-terminal domain of GSDMB interacts with key components of the autophagy machinery, including LC3B and Rab7, thereby activating Rab7 during pro-survival autophagy in response to anti-HER2 treatment. Furthermore, the lack of GSDMB appears to significantly impede Rab7-mediated maturation of autophagosomes (Gámez-Chiachio et al. 2022). Therefore, CRISPR system provides a great platform for targeting GSDMS in order to regulate autophagy in cancer treatment.

In addition to using CRISPR-Cas application for targeting autophagy modulators, CRISPR screen has contributed in identification of implicated genes. For instance, CRISPR-Cas9 high-throughput screening elucidated a radioresistant-related gene *LUC7L2* that encodes the RNA-binding protein LUC7L2 (Shen et al. 2021; Li et al. 2021). Downregulation of *LUC7L2* in radioresistant nasopharyngeal carcinoma cells leads to suppression of sequestosome 1 (SQSTM1 or p62), expression and elevation of autophagy level. Moreover, knockdown of *LUC7L2* combined with autophagy inhibitor, chloroquine (CQ), increased cell death in radioresistant nasopharyngeal carcinoma (Shen et al. 2021).

The high autophagy levels are frequent event in cisplatin-resistant cancer cells which grants survival benefit (Lin et al. 2017; Periyasamy-Thandavan et al. 2008; Gąsiorkiewicz et al. 2021). Noteworthy, DNA methylation is a critical contributor in acquiring chemoresistance to cisplatin. Therefore, using CRISPR-dCas9-Tet1 system for targeting demethylation of nicotinamide nucleotide transhydrogenase (*NNT*) CpG island in A549/DDP cells is established to suppress the autophagy and cisplatin resistance as a first-line chemotherapeutic agent for treting advanced non-small cell lung cancer (Xu et al. 2023).

The prodigy of CRISPR-Cas editing tools is gaining attentions by acting as a scalpel for genomic surveillance. The manifestation of technologies based on the CRISPR-Cas9 has paved the way to define novel pathways and unique factors that are associated with the signaling, recognition, and execution of autophagy via forward genetic screening in mammalian cells (Shoemaker et al. 2019). The trend of genome editing is set to potentially aid to explore the underpinning mechanisms in autophagy studies in detail via genome modification (Cui et al. 2017). Both CRISPR nuclease and CRISPRi are redundant for loss-of-functions studies and both have contributed to autophagy, while CRISPR nuclease is more widely adopted (Shoemaker et al. 2019). Therefore, considering the great contribution of CRISPR-Cas9 in understanding the molecular machineries of various biological function implicated in autophagy process (Cui et al. 2017), this technology offers more than a promise for studying and targeting autophagy in tumor cells.

### Necroptosis: the modern age

Necrotic cell death is a distinct, caspase-independent cell death mechanism characterized by a hallmark of cytoplasmic swelling and organelle disruption, accompanied by compromised cellular membrane integrity and subsequent release of cellular contents into the extracellular environment (Duprez et al. 2009). Apoptosis, as the default cell death cascade, is activated by death receptor ligands in most cell lines. Instead, necrotic cell death is invoked as a back-up pathway if the prosses of caspase activation is impeded (Duprez et al. 2009). An apoptosis/necrosis chimera, the necroptosis cell death takes place through a programmable mechanism with a characteristic necrotic cell death phenotype (Hanson 2016). In fact, necroptosis has been proposed as a “double-edged sword” as it can trigger an unrestrained inflammatory pathway response, leading to disease chronicity, severe tissue injury, tumor progression, and is also functioning as a host defense mechanism, exerting antitumor and antipathogenic effects via its powerful pro-inflammatory properties (Ye et al. 2023). Chronic necroptosis contributes to tumor necrosis and metastasis, thereby, therapy-induced necroptosis leads to antitumor immunity and tumor suppression (Yan et al. 2022).

Necroptosis can be activated by various stimuli that can trigger the activation of the pseudokinase MLKL (mixed lineage kinase domain-like) and the protein kinase RIP3 (receptor-interacting protein 3). Specifically, activation of necroptosis is contingent upon the interaction between the RIP homology interaction motif (RHIM) and unique proteins containing this motif, such as RIP1, TRIF, and DAI. These RHIM-containing proteins serve as transducers, transmitting signals from necrotic activators to mediators RIP3-MLKL, ultimately leading to the execution of cell death. Besides, RIP1 serves a critical role in regulation of necroptotic cell death (Almagro and Vucic 2015). This occurs particularly when either caspase-8 or cFLIP is absent or inactive (Maji et al. 2024).

**Fig. 5:**
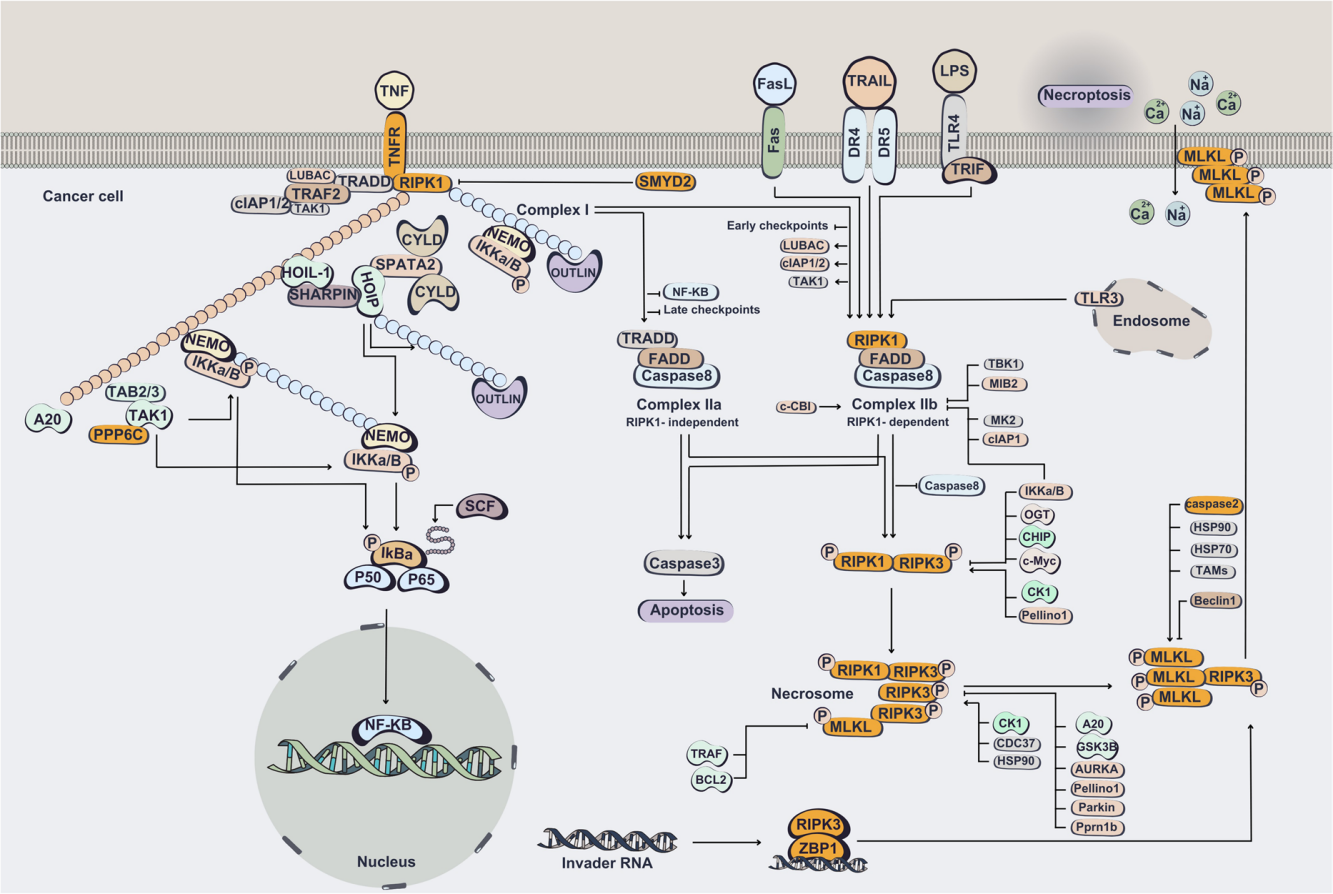
CRISPR-Cas targeting of necroptosis pathway in cancer. The components that have been targeted by CRISPR system in cancer cells are represented in yellow. TNF ligation induces the formation of complex I, resulting in the activation of the NF-κB signaling pathway. Inhibition of NF-κB results in formation of complex II a containing caspase-8, which induces apoptosis through the cleavage of downstream molecules. The inhibition of RIPK1 ubiquitination or phosphorylation results in formation of complex II b which induces apoptosis through activated caspase-8. When caspase-8 is inhibited, interaction of RIPK1 and RIPK3 results in phosphorylation and oligomerization of MLKL and necrosome formation. MLKL translocation towards the plasma membrane in order to form a pore leads to necroptotic cell death through ion influx, cell swell, and membrane lysis followed by the release of intracellular components. Death ligands (e.g., FasL and TRAIL) trigger necroptosis through inducing the formation of necrosome complex. LPS, poly(I:C), double-stranded and viral RNAs trigger necroptosis through the formation of TRIF-mediated necrosome complex. Viral or cellular endogenous RNAs bind to ZBP1 and ZBP1–RIPK3 complex form, leading to RIPK1-independent necroptosis

Whereas, its molecular machinery has been well explored, the exact process of regulation and function of tumor cells necroptosis in tumorigenesis and metastasis is complex and just recently began to emerge (Yan et al. 2022). So far, the application of CRISPR tools in the concept of necroptosis has been mostly limited to identification of regulatory and implicated components. Using CRISPR screens in KBM7 and HAP1 cells has identified key signaling mediators in necroptosis, such as RIP kinase 1 and 3 (RIPK1 and 3), TNFR1, and MLKL (Fauster et al. 2019) (Fig. 5).

In another attempt, CRISPR-Cas9 screen of all targetable mouse protein coding genes for necroptosis resistance in L929 mouse fibroblast cells.

several key regulators were identified, including the TNF receptor *Tnfrsf1a* and components of the necrosome, *MLKL* and *RIPK1/3*. This study also established CYLD, a deubiquitylase specific to RIPK1, as a positive regulator of necroptosis, consistent with its ability to facilitate the translocation of RIPK1 from the TNFR1 complex to the necrosome. Furthermore, SPATA2 was found to be responsible for recruiting CYLD to the TNF receptor complex, highlighting its critical role in modulating necroptotic signaling (Callow et al. 2018).

Through the application of a sensitized CRISPR whole-genome knockout screen, it has been discovered that protein phosphatase 1 regulatory subunit 3G (PPP1R3G) is indispensable for RIPK1-dependent apoptosis and type I necroptosis. This finding suggests that the PPP1R3G/PP1γ complex represents a promising therapeutic target for modulating RIPK1 perturbation-associated human diseases, highlighting its potential as a key regulator of these pathophysiological processes (Du et al. 2021). Moreover, a comprehensive genome-wide CRISPR-Cas9 library screen has revealed that protein phosphatase 6 catalytic subunit (PPP6C) plays a critical role in elevating TNF-induced necroptosis. Furthermore, depletion of PPP6C was found to protect cells from TNF-induced necroptosis in a phosphatase-activity-dependent manner. Interestingly, PPP6C acts as a phosphatase for TGF-β activated kinase 1 (TAK1), inhibiting its kinase function. Deletion of *PPP6C* leads to hyperactivation of TAK1 and impaired RIPK1 kinase activity upon TNF stimulation. Notably, heterozygous deletion of *PPP6C* was found to improve necroptosis-related tissue injury and inflammation in the mouse gastrointestinal tract. Collectively, these findings suggest that PPP6C is a crucial regulator of necroptosis and highlight the central role of phosphatases in modulating necroptosis-related diseases (Zou et al. 2022).

The expression of pivotal modulators of the necroptotic cascade is generally suppressed in cancer cells, indicating that cancer cells might avoid necroptosis to survive (Tong et al. 2022). For instance, downregulation of RIPK3 has been reported in acute myeloid leukemia (AML) (Höckendorf et al. 2016), colorectal cancer (Feng et al. 2015), breast cancer (Stoll et al. 2017; Koo et al. 2015), and melanoma patients (Höckendorf et al. 2016). In fact, loss of RIPK3 expression has been established as a common feature of necroptosis escape in some cancer cells (Najafov et al. 2017). Generation of human and mouse Ubiquitin‐specific peptidase 22 (*USP22*) KO or *RIPK3* KO cells using CRISPR-Cas9 suggested that USP22 controls necroptosis by regulating RIPK3 ubiquitination (Roedig et al. 2021). Furthermore, targeting RIPk1/3 using CRSIPR-Cas9 system in cholangiocarcinoma established that targeting *RIPK1* results in suppressed TLR3 ligand and Smac mimetic induced apoptosis and necroptosis (Lomphithak et al. 2020). RIPK1 has been shown to regulate both caspase-8-dependent apoptosis and RIPK3 and MLKL-dependent necroptosis (Lin et al. 2016). Additionally, RIPK1 can suppress apoptosis and necroptosis in a kinase-independent manner. In mouse models, RIPK1 deficiency or mutations that disrupt its RHIM domain trigger ZBP1-dependent necroptosis and inflammation. ZBP1 is a key mediator of interferon-induced necroptosis, which is activated through RIPK3 (Malireddi et al. 2019). The deletion of ZBP1 in MVT-1 cells has been demonstrated to block tumor necroptosis during tumor development and suppress metastasis, suggesting that ZBP1 plays a role in mediating tumor necroptosis during tumor development in preclinical cancer models (Baik et al. 2021). Furthermore, RIPK1 is established as a target of the SET and MYND domain-containing protein 2 (SMYD2) (Malireddi et al. 2019). SMYD2 is a histone lysine methyltransferase which has been implicated in carcinogenesis and inflammation (Zhou et al. 2023b). As well it is identified to promote TNF-induced apoptosis and necroptosis (Malireddi et al. 2019). In that regard, in vivo and in vitro KO of SMYD2 using CRISPR-Cas9 is shown to sensitize colonic tumor cells to apoptosis and necroptosis induced by TNF (Yu et al. 2022).

As alluded in Fig. 5 caspases participate in necroptosis process (Hanson 2016). Adopting a CRISPR-Cas gene editing system for targeting of caspase-9 in mice results in impaired association of RIPK1/3 and suppressed phosphorylation of RIP kinases, whereas the upregulation of RIPK1 or RIPK3 rescued the effect of caspase-9 deficiency (Molnár et al. 2021). Noteworthy, CRISPR-Cas9 targeting of *caspase-2* in human ovarian carcinomas cells enhanced phosphorylation of RIPK1 and MLKL, indicating caspase-2 as a negative regulator of necroptotic cell death to be an important candidate in therapeutic applications (Zamaraev et al. 2018).

Overall, CRISPR-Cas tools have served perfectly in identifying the molecular bases of necroptosis pathways in various cancers, and introducing the potential therapeutic targets, precisely in those that circumvent necroptosis. However, more investigations are required to find the outcome of using CRISPR gene editing technology for battling cancer in term of necroptosis regulation.

### Pyroptosis: a deal breaker

**Fig. 6:**
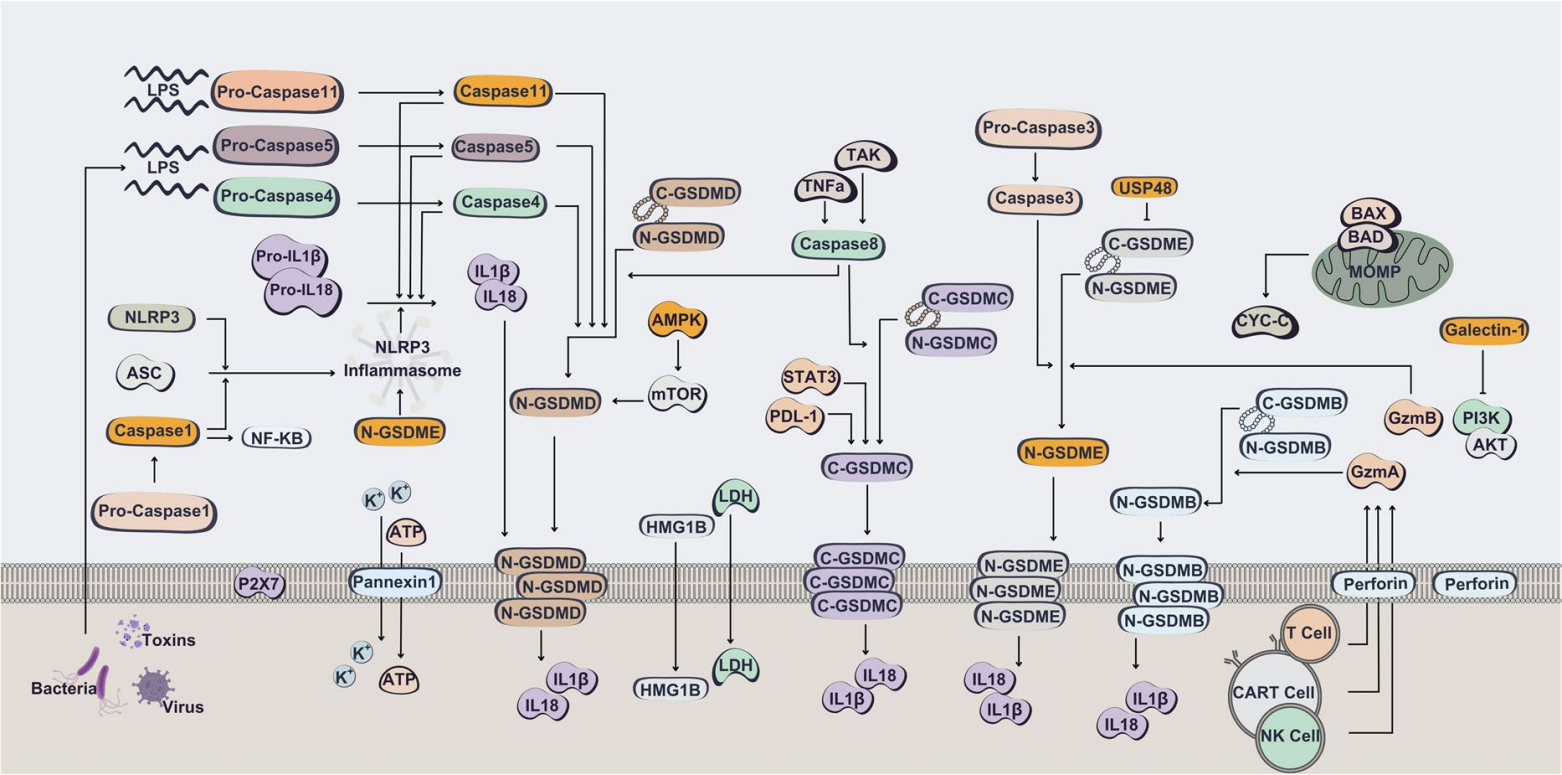
CRISPR-Cas targeting of canonical inflammasome and non-canonical inflammasome pathway in cancer cell pyroptosis. In the canonical inflammasome in response to exogenous pathogens and endogenous damage, ASC recruit pro-caspase 1, leading in activation of caspase 1 which in complex with ASC and NLRP3 form the NLRP3 inflammasome and subsequently cleaves pro-IL-1β, pro-IL-18, and GSDMD protein. Liberation of the GSDMD cytotoxic N-terminus results in formation of a pore on plasma membrane to release mature IL-1β, IL-18. In non-canonical pathway, LPS from extracellular Gram‐negative bacteria directly binds to murine pro-caspase 11 or its human homologs pro-caspase 4 and 5, resulting in activation of caspase 11/4/5. In this pathway, cleavage of GSDMD occurs by caspase 11 or 4/5 upon direct binding of cytosolic LPS. In pyroptosis induced by chemotherapy, pyroptosis in epithelial cells occurs through activating mitochondrial death machinery and caspase 3, which cleaves GSDME. GSDME-N in turn activates NLRP3 inflammasome, and subsequently activates caspase 1/GSDMD cascade. Cleavage of gasdermins could occur by Lymphocyte-derived granzymes proteases, to trigger pyroptosis of cancer cells. The components that have been targeted by CRISPR system in cancer cells are represented in yellow

Pyroptosis is a type of programmed cell death that is modulated by gasdermins and is a product of constant cell expansion until the cytomembrane degrades, leading to the release of cellular components that can trigger robust inflammatory and immune responses. Pyroptosis, an innate immune response, could be activated by inflammasomes via several influencing factors. The inflammasomes activation can promote the maturation of caspase-1 or -4/5/11 (Wei et al. 2022; Wang et al. 2018). Using a CRISPR-Cas9 screen of caspase-11- and -1- -mediated pyroptosis in mouse bone marrow macrophages, has revealed that the caspase-1 or -4/5/11 can cleave gasdermin D (GSDMD) to release its N-terminal domain (Shi et al. 2015), that can attach membrane lipids and perforate the cell membrane (Fig. 6) (Wei et al. 2022; Wang et al. 2018). Furthermore, using CRISPR-Cas9 system for targeting *AMPKα1* or *AMPKα2* in HEK293 has led to identifying that GSDMD-mediated pyroptosis is under the regulation of AMPK-mediated phosphorylation in tumor cells (Chu et al. 2023).

Gasdermin is identified as the executor of pyroptosis and gasdermin-mediated cancer cell pyroptosis has become an essential and frontier field in oncology. Induction of cancer cell pyroptosis has been demonstrated that can heat tumor microenvironment and thereby elicit the robust anti-tumor immunity to inhibit tumor growth (Hou et al. 2023). In fact, pyroptosis can inhibit tumor development and promote an antitumor immunity, and triggering pyroptosis is a promising therapeutic strategy for cancer (Ren et al. 2023). GSDME is a mediator of cancer cell pyroptosis dependent on caspase-3 cleavage (Wang et al. 2018). It is established that melanoma cells with GSDME defects create larger tumors than wild-type melanoma cells; thereby, GSDME might have anti-tumor activity (Rogers et al. 2019). A CRISPR-Cas9 screen approach for identifying regulators of pyroptosis in cancer revealed that loss of USP48, a deubiquitinating enzyme, remarkably suppressed cell pyroptosis. USP48 enhanced pyroptosis through stabilizing GSDME. Indicating that USP48 activation to be a sufficient strategy to sensitize cancer cells to pyroptosis and elevates response to immunotherapy (Ren et al. 2023). Furthermore, *GSDME* KO by CRISPR–Cas9 in SH-SY5Y is shown to block pyroptosis (Zhang et al. 2020). Wang et al. employed a nano-CRISPR scaffold, referred to as Nano-CD, to co-deliver CRISPR/dCas9 and cisplatin to B16F10 melanoma cells. This approach induced pyroptosis, a form of programmed cell death, by triggering the expression of endogenous GDSME and activating caspase-3-mediated immunogenic cell death (Wang et al. 2023a). Moreover, *GSDME* KO by CRISPR-Cas9 in SGC-7901 gastric cancer cells switched drug-induced caspase-3 dependent apoptosis into pyroptosis (Wang et al. 2018).

Mending evidence have suggested that caspase-11 is a great contributor in pyroptosis process (Ye et al. 2019; Abu Khweek and Amer 2020). Moreover, CRISPR-Cas system has served greatly for targeting *caspase-11* in cancer therapy. Generation of *caspase11*-deficient MS1 cells and *caspase-4*-deficient HeLa cells using CRISPR–Cas9 resulted in reduced galectin-1 release upon LPS electroporation (Russo et al. 2021). Galectin-1, a carbohydrate-binding protein, is released during pyroptosis and is a great contributor to tumor transformation, apoptosis, cell cycle regulation, and inflammation (Russo et al. 2021; Rabinovich 2005). CRISPR-Cas9 KO *GALECTIN-1* in head and neck squamous cell carcinoma models (MOC2 and MEERL) leads to reduced tumor growth by ~ 50%. Furthermore, *GALECTIN-1* knockout cells secreted limited levels of monocytes and macrophage recruiting chemokines including G-CSF, CCL5, and CXCL1. Analyzing the gene expression signatures of wild type and KO *MOC-2* tumor cells also revealed the differences of pivotal signaling cascade, such as downregulation of PI3K-AKT cascade in KO-tumors (Nambiar et al. 2018). Noteworthy, CRISPR-Cas KO of *GALECTIN-1* has been the center of attention for cancer research, particularly in head and neck tumors. These studies have suggested that targeting *GALECTIN-1* a promising approach for cancer treatment as it can contribute to tumor metastasis and apoptosis (Nambiar et al. 2017a; Nambiar et al. 2017b), also they highlight the potential of CRISPR approaches in cancer therapy.

It is essential to consider that pyroptotic death is an inflammatory type of cell death and deregulation of inflammasome activation can contribute to cancer development (Faria et al. 2021). A critical signaling cascade resulting in acute and chronic inflammation occurs by the activity of NLRP3 inflammasome tailed with caspase-1-dependent release of IL-1β and -18 pro-inflammatory cytokines, along with GDSMD-mediated pyroptotic cell death (Faria et al. 2021). It has been suggested that NLRP3 inflammasome signaling is related to carcinogenesis and that suppression of NLRP3 inflammasome could serve as a cancer prevention strategy. Hence, genomic editing tools such as CRISPR-Cas9 are great strategies for directly targeting NLRP3 due to their extreme effectiveness, specificity, and simplicity. It has been proposed that breast cancer may be treated by targeting the NLRP3 inflammasome through gene editing (Wang et al. 2023b). NLRP3 deficiency by CRISPR-Cas9 in SK-Hep1 hepatocellular carcinoma cells results in delayed cell growth, suppression of PI3K, p-AKT, pNF-κB N-cadherin, and MMP-9, as well as cell cycle arrest at G1 phase through an increase in p21 and a reduction in CDK6 (Choi and Cho 2021). CRISPR–Cas9-generated NLRP3-KO THP-1 cells and mice established that myeloid PTEN can regulate the responsiveness of chemotherapy by enhancing NLRP3-dependent antitumor immunity and suggest that myeloid PTEN as a promising biomarker for prediction of chemotherapy responses (Huang et al. 2020).

Overall, different cancer cells trigger pyroptosis in various routes with different effects in different cancer backgrounds. Pyroptosis exerts complicated effects on cancer development, such as suppression of cancer cell viability, improvement of antitumor immunity, altering invasion and migration of cancer cells, and enhancement of chemotherapy sensitivity (Huang et al. 2022). Demonstrating the mechanisms of the multifaceted effects of pyroptosis contributes to identification of treatment approaches for cancer in the future (Huang et al. 2022). In that regard, considering the wide use of CRISPR tools for understanding pyroptosis regulation in cancer cells and identification of suitable targets, this system offers a great promise in developing a personalized cancer therapy based on regulating pyroptosis.

### Ferroptosis: the *iron* soldier

At the heart of tumorigenesis lies the capability of cancer cells to evade programmed cell death, thereby enabling unlimited replication and immortality. Notably, ferroptosis represents a distinct form of PCD that relies on lipid peroxidation, distinguishing it from traditional other PCD forms in terms of its morphology, physiological characteristics, and biochemical mechanisms (Wang et al. 2020).

**Fig. 7:**
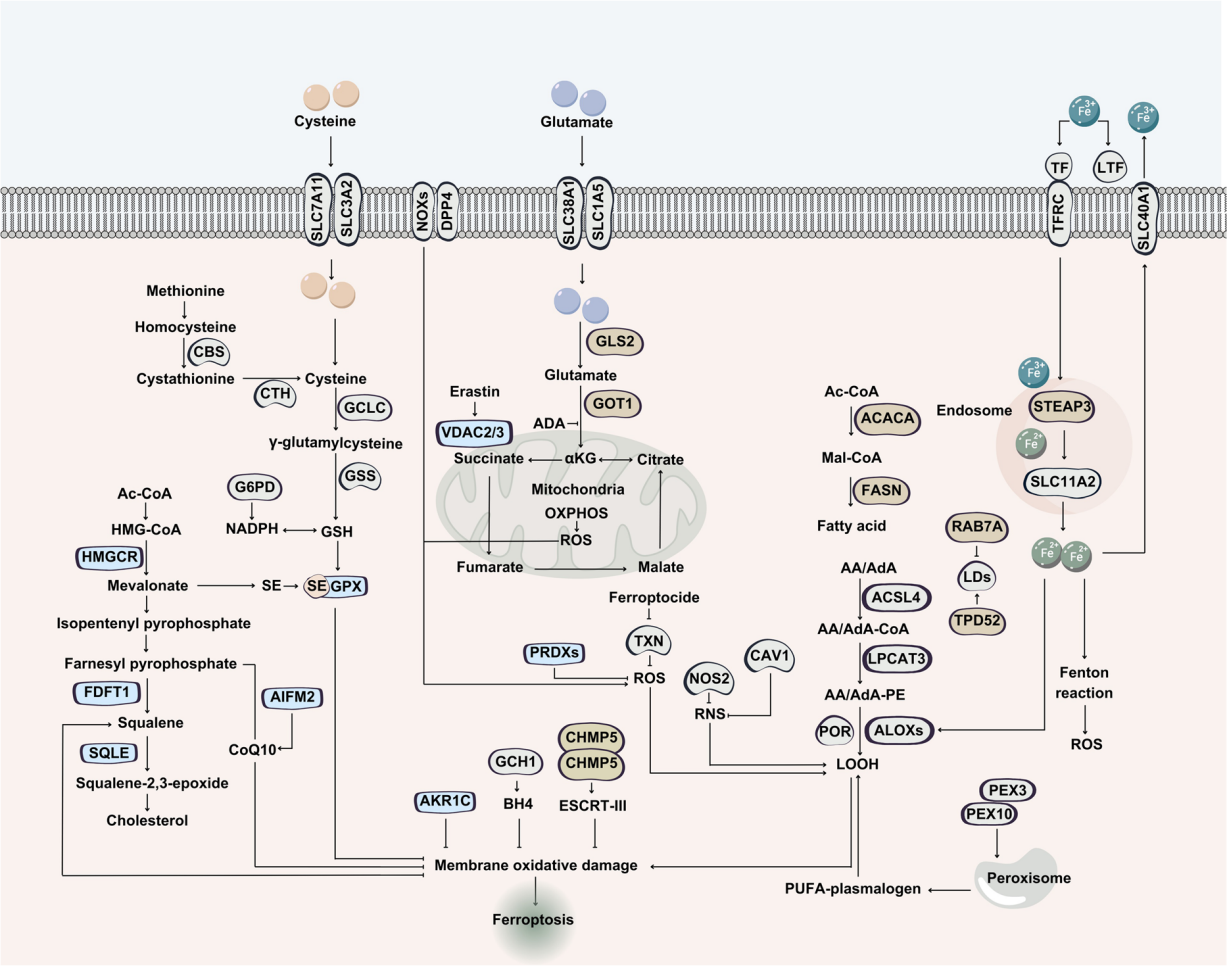
Signaling pathway implicated in ferroptosis cell death. Ferroptosis is distinguished by the presence of abnormally small mitochondria with compacted mitochondrial membrane densities, diminished or absent mitochondrial cristae, and ruptured outer mitochondrial membranes. This mode of cell death can be triggered by both experimental compounds, such as erastin, Ras-selective lethal small molecule 3, and buthionine sulfoximine, as well as clinically approved drugs, including sulfasalazine, sorafenib, and artesunate, in both cancerous and normal cells

Ferroptosis arises from an imbalance in lipid peroxidation, triggered by the accumulation of reactive oxygen species (ROS) that surpasses the reducing capacity of glutathione (GSH) and GPX4. Lipids, iron, and ROS are crucial for cell survival in normal physiological conditions, but their excessive dependence can have devastating consequences (Fig. 7). When metabolic disorders occur, these very same factors that maintain homeostasis in a steady state can become a deadly force against cells (Zhao et al. 2022).

The energy metabolism pathways are established to play a pivotal role in modulating ferroptosis. In fact, tumor metabolism not only creates a microenvironment conducive to metastasis but also inhibits the ferroptotic cascade within the cancer cells via HCAR1/MCT1-SREBP1-SCD1 axis, potentially enabling their metastasis and development. Interestingly, lactate produced by tumors elevated lipid synthesis by suppressing glycolysis, and it also induced the expression of HCAR1 (lactate receptor) and MCT1 (lactate transporter), thereby reinforcing its own production (Zhao et al. 2022).

Notwithstanding the energy stress and lactate signaling pathways triggered by tumor metabolism, the master regulators of cell growth, mTORC1 and mTORC2, have been implicated in the regulation of ferroptosis. Moreover, Hippo, which regulates organ size through altering cell proliferation and apoptosis, and E-cadherin, which maintains cell polarity and contributes to ferroptosis, also play roles in ferroptosis (Zhao et al. 2022). Notably, research has revealed that p53, a key tumor suppressor protein, not only induces apoptosis but also plays a critical role in altering ferroptosis in certain cancer cells (Zhao et al. 2020).

It has been demonstrated that ferroptosis can effectively impede the growth of malignant cells in various cancers, including liver, pancreatic, prostate, and breast cancers. Moreover, aggressive cancer cells have been found to be inherently susceptible to ferroptosis, sparking interest in exploiting this process as a novel therapeutic strategy for cancer therapy (Zhao et al. 2020). In a recent study, Valashedi et al. utilized the CRISPR/Cas9 gene editing tool to selectively target the antioxidant-related protein Lipocalin 2 (Lcn2), which is overexpressed in cancer cells. The results demonstrated that silencing *Lcn2* expression inhibited cell proliferation and migration, and significantly increased the sensitivity of MDA-MB-231 breast cancer cells to cisplatin treatment. Furthermore, downregulation of Lcn2 led to an enhancement of erastin-induced ferroptosis in MDA-MB-231 cells (Valashedi et al. 2022). Erastin is a pivotal activator of ferroptosis, as it can trigger several molecules (Zhao et al. 2020). Moreover, Chen et al. used CRISPR-based loss-of-function genetic screens for identification of chemotherapy-related synthetic lethal genes in HCC cells. They established that inhibiting phosphoseryl-tRNA kinase (PSTK) increases the sensitivity of HCC cells to chemotherapy. Notably, PSTK was found to suppress ferroptosis induction in HCC cells in response to chemotherapy, whereas its depletion led to the inactivation of glutathione peroxidase 4 (GPX4) and glutathione metabolism, ultimately elevating ferroptosis induction upon targeted chemotherapy treatment. Notably, punicalin, a hepatitis B virus (HBV) treatment agent, was identified as a potential PSTK inhibitor that showed synergistic efficacy when combined with Sorafenib in vitro and in vivo HCC treatments (Chen et al. 2022).

Using a whole-genome CRISPR/Cas9 screen, researchers have identified key intrinsic mechanisms of ferroptosis resistance in HCC cells. The study found that tripartite motif-containing protein 34 (TRIM34) plays a crucial role in decreasing ferroptosis in HCC cells, suggesting its potential as a therapeutic target to enhance sensitivity to ferroptosis-inducing treatments. Targeting TRIM34 was established to enhance ferroptosis sensitivity and enhances the efficiency of immunotherapy in HCC. YY1 upregulates TRIM34, which promotes ubiquitin-mediated degradation of UPF1, thus promoting GPX4 expression and is associated with poor prognosis. Therefore, targeting TRIM34 appears as potential strategy for HCC treatment (Yao et al. 2024).

A genome-wide CRISPR/Cas9 knockout screening was conducted in HepG2 and SK-Hep-1 cell lines to identify key regulators of ferroptosis in HCC cells. The study found that cAMP response element-binding protein (CREB) regulated transcription coactivator 3 (CRTC3) plays a protective role against ferroptosis induced by chemotherapeutic agents and also reduces the efficacy of interferon-gamma (IFN-γ) treatment in HCC. Mechanistically, the knockout of CRTC3 alters lipid profiles in tumor cells, leading to an increase in polyunsaturated fatty acids (PUFAs), which enhances lipid peroxidation and makes HCC cells more susceptible to ferroptosis-inducing agents (Li et al. 2023b). Additionally, a genome-wide CRISPR-Cas9 suppressor screen revealed that porphorin (POR) is essential for ferroptotic cell death in cancer cells (Zhang et al. 2022). Additionally, using a Genome-wide CRISPR activation screen in lung cancer cells (KP4 cells) have identified BRM as a ferroptosis suppressor. In fact, CRISPRa screens established that the SWI/SNF ATPase BRM/SMARCA2 is a suppressor of ferroptosis (Bhat et al. 2024). A very recent study by Zhang et al. using genome-wide CRISPR-Cas9 activation and kinase inhibitor library screening approach was employed to identify novel regulators of ferroptosis in AKT-driven cancers. This study revealed that transient receptor potential muclipin 1 (TRPML1) is a promising target for modulating ferroptotic processes in these tumors. Inactivation of the TRPML1 gene or disruption of its interaction with ARL8B protein has been demonstrated to inhibit cancer cell growth both in vitro and in vivo, and enhance sensitivity to ferroptosis. Furthermore, a synthetic peptide targeting TRPML1 was found to suppress cancer cell proliferation and improve the efficacy of cancer treatment by sensitizing tumor cells to therapy (Zhang et al. 2024).

A recent study by Takahashi et al. used a combination of 3D cancer spheroid models and CRISPR-Cas9 screens to investigate the molecular mechanisms underlying the pathogenesis mediated by NRF2 hyperactivation. In lung tumor spheroids, the study found that NRF2 hyperactivation is essential for both proliferation and survival of cells. The CRISPR screens revealed that mTOR is required for proliferation in these spheroids, while GPX4 is necessary for protecting inner cells lacking a matrix from ferroptosis. Furthermore, the study showed that ferroptosis inhibitors prevented cell death caused by NRF2 downregulation, highlighting the critical role of NRF2 in protecting matrix-deprived cells from ferroptosis (Adamiec-Organisciok et al. 2024). A study by Zou et al. has shown that depleting *HIF-1α* using CRISPR technology significantly reduces the sensitivity of ES-2 cells to ferroptosis. The researchers found that the HIF pathway plays a central role in determining ferroptosis susceptibility in clear-cell carcinomas, suggesting that targeting this pathway may be a potential therapeutic strategy for these cancers (Zou et al. 2019). A recent study employed a CRISPR-Cas9 library screen to identify epigenetic factors that regulate ferroptosis inhibition. The results of this study identified coactivator-associated arginine methyltransferase 1 (CARM1) as a crucial regulator of ferroptosis inhibition. Depletion of *CARM1* in cells treated with Sorafenib led to enhanced ferroptosis, characterized by reduced cell viability, decreased glutathione levels, increased lipid peroxidation, and altered mitochondrial cristae structure. These findings suggest that CARM1 plays a critical role in modulating ferroptosis resistance in cells treated with Sorafenib (Cheng et al. 2023).

1. In summary, these studies highlight the potential of CRISPR-based approaches for investigating the role of ferroptosis in cancer cells and for targeting this programmed cell death pathway as a promising therapeutic strategy for cancer treatment.

## Today's perspective and the land of tomorrow

The relationship between cell death and cancer is becoming increasingly complex, necessitating the development of advanced tools to study cancer cell death resistance. (Labi and Erlacher 2015). CRISPR technology offers a powerful platform for identifying relevant genes, generating modified models, and eradicating resistance through genetic manipulation. However, successful CRISPR screening relies on several factors, including optimized biological models, gene perturbation, calibrated stimuli, and read-outs. Efficient delivery of CRISPR components, particularly in vivo and in primary cells, is a significant challenge that requires careful optimization. (Shirani-Bidabadi et al. 2023). Furthermore, advancements in delivery methods are necessary to enable in vivo application (Bock et al. 2022a). To achieve translational medicine, it is essential to develop Cas9 variants with minimal off-target effects and methods to enhance precision through homology-directed repair (Zhan et al. 2019).

While the CRISPR-Cas9 system has shown great promise in cancer therapy, it is essential to acknowledge the various challenges and limitations associated with its use. The potential for off-target effects, where the guide RNA binds to unintended locations in the genome, can lead to unwanted mutations and undermine the efficacy of treatment (Katti et al. 2022; Awwad et al. 2023). Furthermore, the immune response triggered by the Cas9 enzyme may result in inflammation and potential harm to healthy tissues. Additionally, delivery limitations, specificity issues, and the risk of immortalization of cancer cells are all concerns that must be addressed. Moreover, the potential for insertional mutagenesis, limited understanding of off-target effects, and ethical concerns surrounding germline editing are all critical considerations (Fig. 2). Regulatory hurdles, high costs, and the risk of resistance development also pose significant barriers to the widespread adoption of CRISPR-based therapies. As such, it is crucial that continued research and development focus on overcoming these challenges to ensure the safe and effective use of CRISPR-Cas9 in cancer therapy (Barrangou and Doudna 2016; Yin et al. 2019; Zhong et al. 2024).

The development of next-generation CRISPR tools, such as base and prime editing, offers enhanced precision in genome modification, eliminating the risk of unintended DNA double-stranded breaks (Geurts and Clevers 2023). Prime editors (PEs) can induce specific base changes and small insertions/deletions without the need for exogenous DNA templates or double-stranded breaks(Liu et al. 2021). With five generations of PEs, each with improved efficacy, this technology holds potential for individualized therapy (Sen et al. 2023). Prime editing has also been successfully applied in organoids, demonstrating its potential for individualized therapy (Schene et al. 2020). In fact, advances in cancer models and gene editing technologies are required to pave the way of understanding the complexity of cell death regulation in cancers. Complete cell death resistance along with unlimited proliferation in a very short period of time results in tumor growth to a mass of unbearable size, which is not consistent with the usual long latency of malignant disease (Labi and Erlacher 2015).

While the notion that cell death prevents tumor development is widely accepted, it is essential to recognize that cells are constantly replaced and interact with their microenvironment (Labi and Erlacher 2015). This highlights the need for advanced biological models that capture the intricate relationships between the tumor and its surroundings. Organ-on-a-Chip (OoC) technology, which involves growing natural or engineered tissues within microfluidic chips, allows for control over cell microenvironments and preservation of tissue-specific functions (Leung et al. 2022). When combined with CRISPR-Cas technology, OoC enables modeling of complex genetic diseases and environmental factors (Fu et al. 2021). Three-dimensional (3D) bioprinting, which involves depositing living cells and biological materials to create functional tissues or organs, has also been developed (Xu et al. 2022). CRISPR-Cas9 technology can be integrated with OoC and 3D bioprinting to correct endogenous mutations and alter cell function or fate. This approach has shown promise in gene therapy and requires further investigation (Fu et al. 2021).

The CRISPR-Cas9 system has revolutionized cancer research by enabling precise genetic engineering in human cancer organoids (Zhan et al. 2019). However, traditional CRISPR-Cas9 methods have limitations, including the lack of a robust knock-in approach. To overcome this, CRISPR-Cas9-mediated homology-independent organoid transgenesis (CRISPR-HOT) has been developed, allowing for precise integration of exogenous DNA sequences without disrupting TP53 (Artegiani et al. 2020). Combining CRISPR-HOT with other technologies, such as 3D bioprinting and Organ-on-a-Chip, has enabled the creation of highly accurate cancer models for studying complex biological processes and improving therapeutic strategies (Qu et al. 2021).

The vast complexity of cell death pathways, involving numerous genes and mechanisms, necessitates the application of advanced technologies to effectively counteract death resistance in cancer cells. The ability of cancer cells to evade chemo- and radio-therapy by circumventing cell death pathways contributes to tumor progression, metastasis, and ultimately, increased mortality (Kurnit et al. 2017). The emergence of personalized cancer therapy has transformed treatment strategies, enabling individualized molecular analysis to elevate patient care, particularly in advanced and incurable cancers (Zhan et al. 2019). The development of CRISPR technology has significantly accelerated cancer research and holds great promise for identifying mutations and manipulating cell death pathways to improve treatment outcomes. However, despite the rapid progress in this field, further investigations are required to develop an effective and personalized therapeutic approach for targeting cell death pathways using CRISPR technology, leveraging its groundbreaking genetic engineering capabilities.

## Conclusion

Dysregulation of cell death pathways is a fundamental hallmark of cancer development. CRISPR technology has revolutionized cancer therapy by enabling precise and efficient reprogramming of cells to promote cellular death. The rapid evolution of CRISPR tools has enabled significant advancements in personalized cancer therapy. This article aims to highlight the most promising and innovative applications of CRISPR in personalized cancer therapy by targeting programmed cell death pathways, while also discussing the limitations that must be overcome and future perspectives on upcoming advances.

## Data Availability

No datasets were generated or analysed during the current study.
